# Efficacy and Safety of Metreleptin in Patients with Partial Lipodystrophy: Lessons from an Expanded Access Program

**DOI:** 10.4172/2155-6156.1000659

**Published:** 2016-03-23

**Authors:** Nevin Ajluni, Moahad Dar, John Xu, Adam H Neidert, Elif A Oral

**Affiliations:** 1 Brehm Center for Diabetes Research and Division of Metabolism, Endocrinology and Diabetes, University of Michigan, Ann Arbor, MI, USA; 2 Greenville Veterans Affairs Health Care Center, Division of Endocrinology and Metabolism, Brody School of Medicine, East Carolina University, Greenville, NC, USA; 3 AstraZeneca, Gaithersburg, MD, USA

**Keywords:** Diabetes, Hypertriglyceridemia, Metreleptin, Partial lipodystrophy

## Abstract

**Objective:**

Patients with lipodystrophy have severe metabolic abnormalities (insulin resistance, diabetes, and hypertriglyceridemia) that may increase morbidity and mortality. Metreleptin is approved by the United States Food and Drug Administration for treatment of generalized forms of lipodystrophy. We aimed to determine the efficacy and safety of metreleptin among patients with partial lipodystrophy using an expanded-access model.

**Methods:**

Study FHA101 (ClinicalTrials.gov identifier: NCT00677313) was an open-label, expanded-access, long-term clinical effectiveness and safety study in 23 patients with partial lipodystrophy and diabetes and/or hypertriglyceridemia with no prespecified leptin level. Metreleptin was administered subcutaneously at 0.02 mg/kg twice daily (BID) at Week 1, followed by 0.04 mg/kg BID at Week 2. Dose adjustments thereafter were based on patient response (maximum dose of 0.08 mg/kg BID). One-year changes in glycated hemoglobin (HbA1c), fasting plasma glucose, triglycerides, alanine and aspartate aminotransferases, and treatment-emergent adverse events (TEAEs) were evaluated.

**Results:**

HbA1c, fasting plasma glucose, and triglycerides were numerically decreased throughout 1 year, with mean (standard error) changes from baseline of −0.88 (0.62)%, −42.0 (22.4) mg/dL, and −119.8 (84.1) mg/dL, respectively, which were greater among patients with higher baseline abnormalities. Liver enzymes did not worsen, and the most frequently observed TEAEs (≥ 10% incidence) were mild to moderate and included nausea (39.1%), hypoglycemia (26.1%), and urinary tract infections (26.1%)—all reported previously. There were no reports of clinically significant immune-related adverse events or new safety signals.

**Conclusions:**

Our clinical observations document the large heterogeneity and disease burden of partial lipodystrophy syndromes and suggest that metreleptin treatment benefits may extend to patients with partial lipodystrophy. Additional studies are needed to confirm these preliminary findings.

## Introduction

Lipodystrophy is a rare disorder characterized by selective loss or absence of adipose tissue with leptin deficiency, ectopic lipid deposition, and severe metabolic abnormalities [1]. Lipodystrophy can be congenital or acquired and its distribution can be generalized or partial, and lipodystrophy phenotypes continue to be characterized [2–4]. Patients with partial lipodystrophy (PL) are more likely to be older and have more variable patterns of fat loss and/or sparing [2].

**Figure 1:**
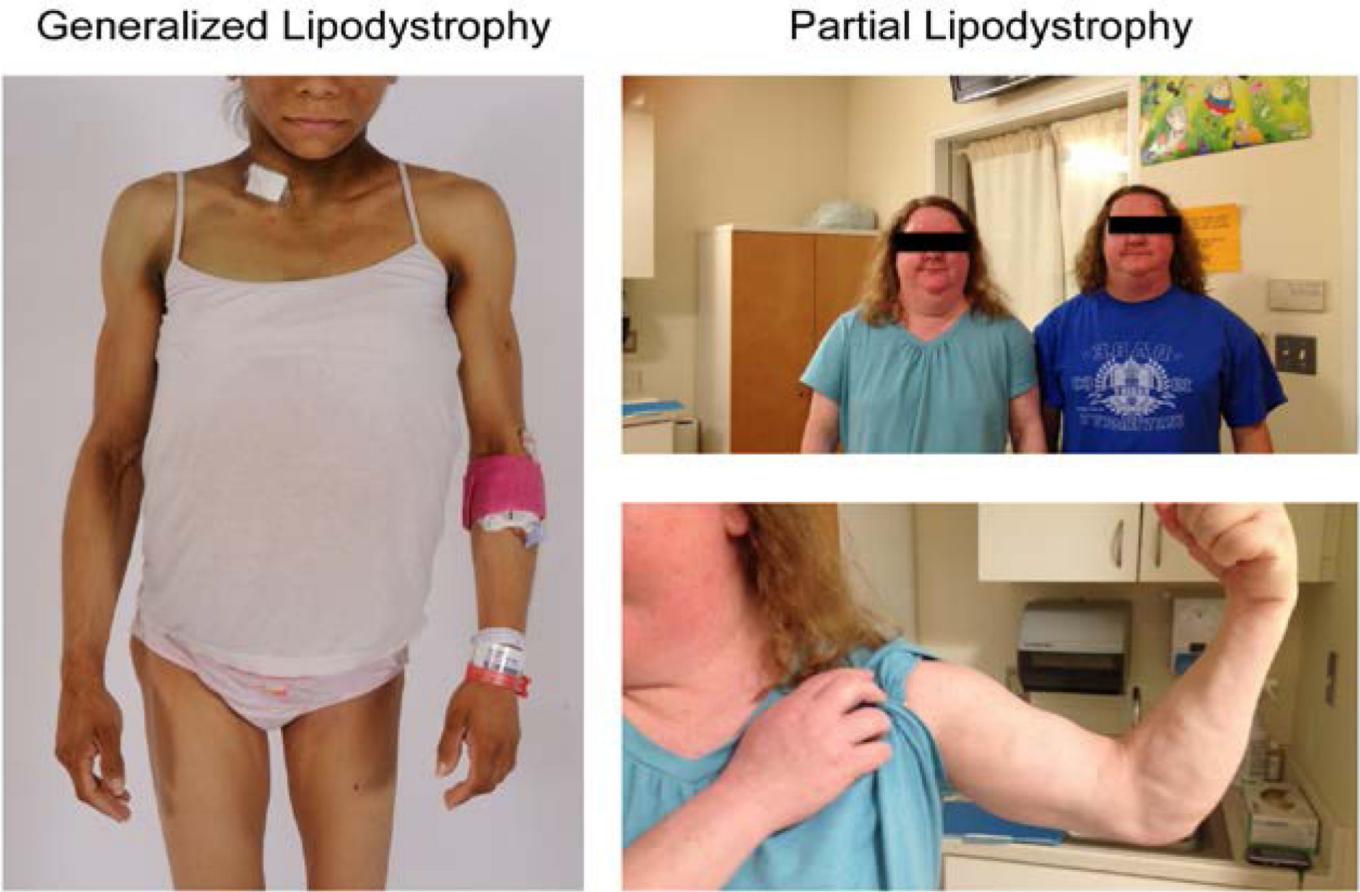
Patients with generalized lipodystrophy and partial lipodystrophy. Left panel: 19-year-old female with acquired generalized lipodystrophy. Right panel: two 40-year-old twin sisters with familial partial lipodystrophy (Patients 20 and 21; Supplemental Table 1), which demonstrates adipose tissue loss in the upper extremities while adipose tissue is preserved in the face and neck.

The physical appearance of patients with familial and acquired PL may be more difficult to discern compared with generalized lipodystrophy (GL) where patients have a stark absence of subcutaneous fat (Figure 1) [2]. Beginning around puberty, patients with familial PL develop loss of adipose tissue in the arms and legs, variable loss in the chest, fat sparing in the face, neck, and abdomen, and increased skeletal muscle volume in the lower extremities [2,5–7]. Patients with acquired PL may experience adipose tissue loss in a cephalocaudal fashion with fat sparing in the lower extremities, starting as early as childhood; however, this pattern and chronology of morphological change are not universal for all patients with this subtype [2,8].

Metabolic abnormalities associated with lipodystrophy include severe insulin resistance, diabetes mellitus, and hypertriglyceridemia [9,10]. Inadequate treatment of these abnormalities can contribute to cardiovascular disease, pancreatitis, and hepatic steatosis, which can increase patient morbidity and mortality [9]. Dietary modification and pharmacotherapy with conventional glucose- and lipid-lowering therapies often prove inadequate at normalizing metabolic disturbances [1]. Metreleptin, a recombinant human analog of leptin, is approved by the US Food and Drug Administration (FDA) as an adjunct to diet as replacement therapy to treat the complications of leptin deficiency in patients with congenital or acquired GL [11].

The use of metreleptin in patients with PL is not currently approved by the FDA or other regulatory authorities. The original cohort of patients reported to be treated with metreleptin included one patient with familial PL [12], followed later by larger cohorts [13–16]. Simha et al. [13] evaluated metreleptin use in patients with PL (Dunnigan variety) who were severely hypoleptinemic (serum leptin range, 0.38–3.69 ng/mL; n=14) and moderately hypoleptinemic (range, 4.1–6.9 ng/mL; n=10). After 6 months of metreleptin treatment, triglycerides and hepatic lipid content were significantly reduced from baseline in both subpopulations, with no significant between-group differences, while glycated hemoglobin (HbA1c) and fasting plasma glucose (FPG) were not significantly decreased. Chan et al. [16] reported improvements from baseline in HbA1c, triglycerides, and liver enzymes from 4 months to up to 3 years among 55 patients with lipodystrophy (GL, n=36; PL, n=19) who were part of an open-ended, open-label study conducted at the National Institutes of Health (NIH). For patients with confirmed metabolic abnormalities at baseline, improvements in HbA1c, triglycerides, and liver enzymes were found to be numerically greater compared to those without baseline abnormalities. In a follow-up analysis that examined 6- and 12-month changes in metabolic parameters with an expanded population set (GL, n=55; PL, n=31), Diker-Cohen et al. [15] reported significant reductions from baseline in HbA1c, FPG, and triglycerides at 12 months among patients with GL and PL, including patients with PL and baseline HbA1c >7.0% or >8.0%, triglycerides >300 or >500 mg/dL, and leptin <4 ng/mL.

Here, we describe the clinical experience and safety of metreleptin treatment among patients with PL who were not required to have a low level of serum leptin as a treatment criterion. The intent of this protocol was to broaden the availability of metreleptin across a wider geographic distribution outside the NIH. The protocol was written solely to make metreleptin available to patients who had a lipodystrophy diagnosis established by a clinical endocrinologist and who were looking to improve quality of life. At the time of conception, the study was not designed to evaluate efficacy with primary hypothesis testing.

## Materials and Methods

Study FHA101 (ClinicalTrials.gov identifier: NCT00677313) was an open-label, sponsor-initiated, expanded-access, long-term safety and efficacy study conducted at multiple treatment centers in the United States among patients with lipodystrophy (mostly PL). Inclusion criteria of this open-access program included male and female patients aged ≥5 years, a clinical diagnosis of acquired or inherited lipodystrophy based on physician diagnosis, and the presence of clinical diabetes based on American Diabetes Association criteria (FPG >126 mg/dL or HbA1c >6.5%) and/or hypertriglyceridemia (>200 mg/dL). Exclusion criteria included the presence of human immunodeficiency virus infection, infectious liver disease, or acquired lipodystrophy with hematologic abnormalities. Study enrollment was based on historical data within the last 3 months of baseline assessment reviewed by a medical physician.

There was no prespecified leptin level to qualify for the study. Study FHA101 was conducted in accordance with the Declaration of Helsinki with protocols approved by an institutional review board at each site. All patients provided written informed consent. This report focuses on the 23 patients with PL from a data cut performed in 2012.

**Figure 2:**
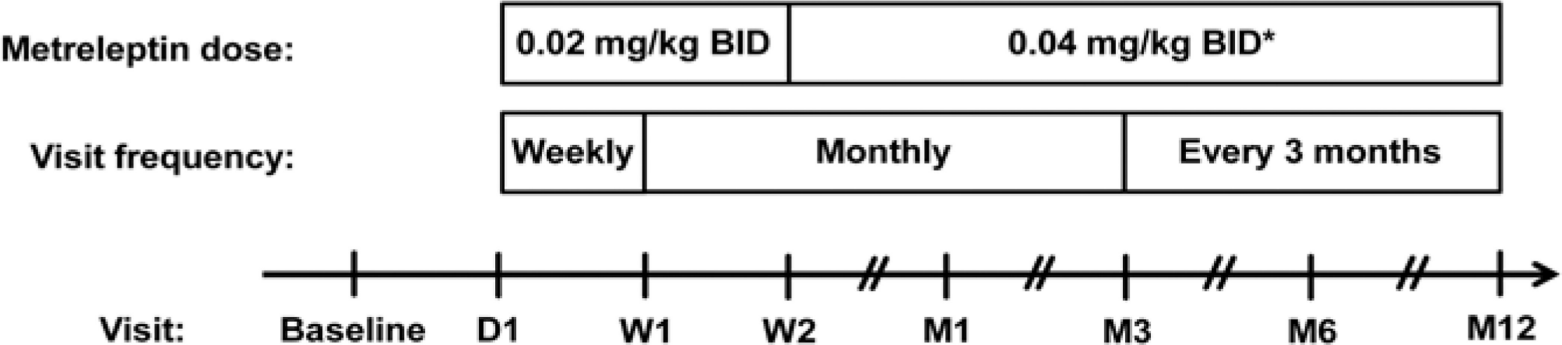
Study design and treatment algorithm after enrollment. BID, twice daily; D, day; M, month; W, week. *Metreleptin dose titration up to 0.08 mg/kg BID was allowed if there were no improvements in metabolic parameters, and a reduction in target dose was permitted if tolerability became an issue.

On Day 1 and after collection of baseline measurements and training, patients, guardians, or caregivers injected metreleptin subcutaneously at 0.02 mg/kg twice daily (BID) for 1 week followed by 0.04 mg/kg BID for the second week (Figure 2). Thereafter, dosage adjustments were allowed based on patient response. Dose titration up to 0.08 mg/kg BID was allowed if there were no improvements in metabolic parameters, and a reduction in target dose was permitted if tolerability became an issue. If metabolic parameters were stabilized after 1 year of treatment, then a decrease in dosing frequency from BID to once daily was allowed. Patients continued concomitant glucose-and lipid-lowering medications after the baseline visit, and further adjustments were permitted at the discretion of the treating physician.

Patients met with their treating physician 1 week after the first treatment and monthly for the first 3 months, followed by every 3 months throughout the first year. At each visit, fasting blood samples for clinical effects and safety measurements were collected, body weight was recorded for dosing, and concomitant medications and adverse events (AEs) were reviewed. Samples for the analysis of anti-metreleptin antibodies were collected at the first treatment visit and at Months 1, 6, and 12 [17].

Clinically relevant parameters included changes from baseline in HbA1c, FPG, and triglycerides throughout 1 year in all patients with PL, in patient subsets with baseline abnormalities (HbA1c ≥8.0%, FPG ≥126 mg/dL, and triglycerides ≥200 mg/dL), and in individual patients. Safety endpoints included changes from baseline in alanine aminotransferase (ALT) and aspartate aminotransferase (AST) and the incidence of treatment-emergent AEs (TEAEs) throughout 1 year.

There was no formal statistical hypothesis or statistical testing prespecified due to the intent of expanded access. Changes from baseline in efficacy endpoints and liver enzymes are presented as means (standard error [SE]) with 95% confidence intervals (CIs) in the intent-to-treat (ITT) population (March 2012 data cut). Baseline demographics and TEAEs are summarized by descriptive statistics in the ITT population.

## Results

**Figure 3:**
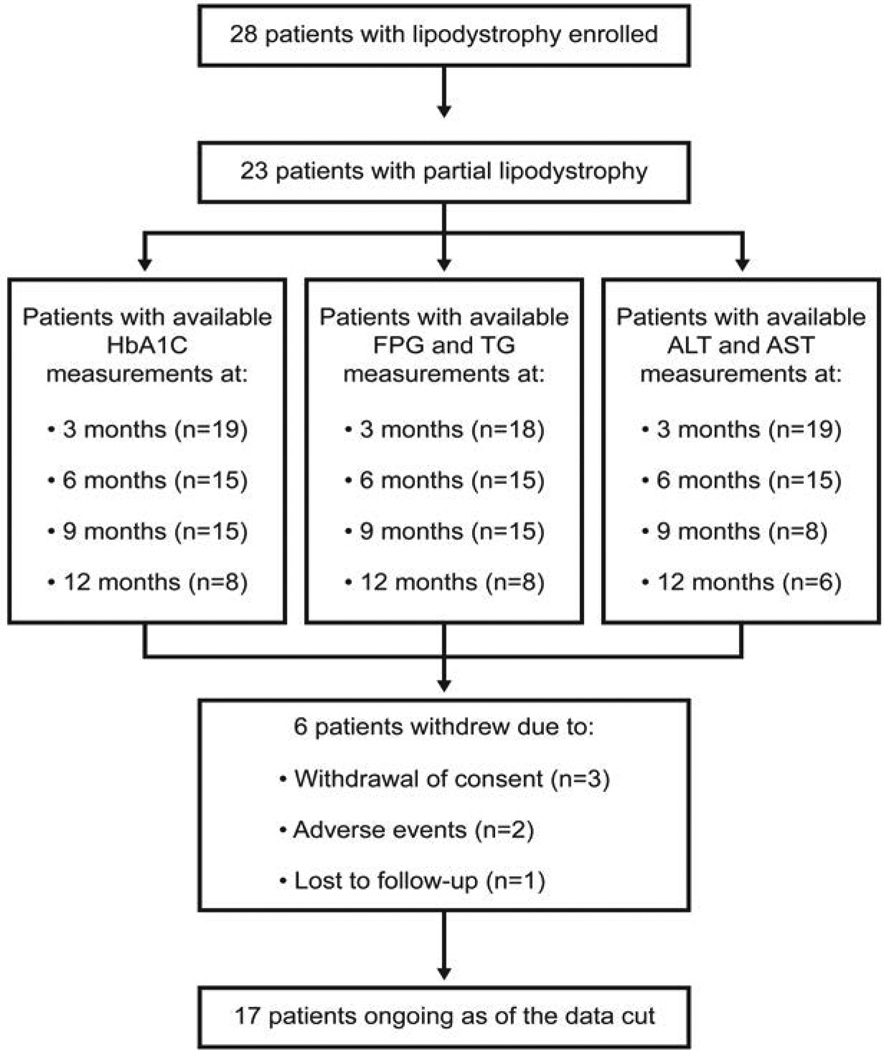
Patient disposition and data availability. ALT: Alanine Aminotransferase; AST: Aspartate Aminotransferase; FPG: Fasting Plasma Glucose; HbA1c: Glycated Hemoglobin; TG: Triglycerides

A total of 23 patients with PL and 5 patients with GL enrolled in the study and received ≥1 dose of study medication as of the March 2012 data cut. Of those 28, 8 patients withdrew (Figure 3). Herein, we report on all 23 patients with PL. Three of the 5 patients with GL treated at the University of Michigan have been reported separately [18].

Most of the 23 PL patients were female and white, with a median age of 47 years and mean (standard deviation [SD]) fasting serum leptin of 14.8 (10.3) ng/mL (Table 1). Patients had multiple comorbidities (hypertriglyceridemia, n=13; hypertension, n=16; diabetes mellitus, n=17; and hepatic steatosis, n=18) and were in need of additional treatments to overcome clinical challenges. Mean (SD) HbA1c and fasting triglyceride levels were 7.9 (1.5)% and 401.9 (537.1) mg/dL, respectively. Since the study was designed to focus on metabolic abnormalities, all but 1 patient had clinically significant diabetes and/or hypertriglyceridemia (described in greater detail in *Safety*). One-half of all patients were taking insulin with or without an oral glucose-lowering agent for diabetes and one-half were taking a fibrate with or without fish oil or a statin for hypertriglyceridemia (Supplemental Figure 1). A detailed listing of concomitant medications for individual patients is provided in Supplemental Table 1. It is important to note that some patients may have reached the 1-year endpoint after the data cut (Table 1).

The number of patients at the time of the 2012 data cut was small, and the number of patients with available measurements for changes from baseline in clinical efficacy measures and liver enzymes decreased over time (Figure 2).

### Clinical effects

The weighted average daily metreleptin dose throughout 1 year in patients with PL was 0.084 mg/kg (range, 0.039–0.134 mg/kg).

**Figure 4:**
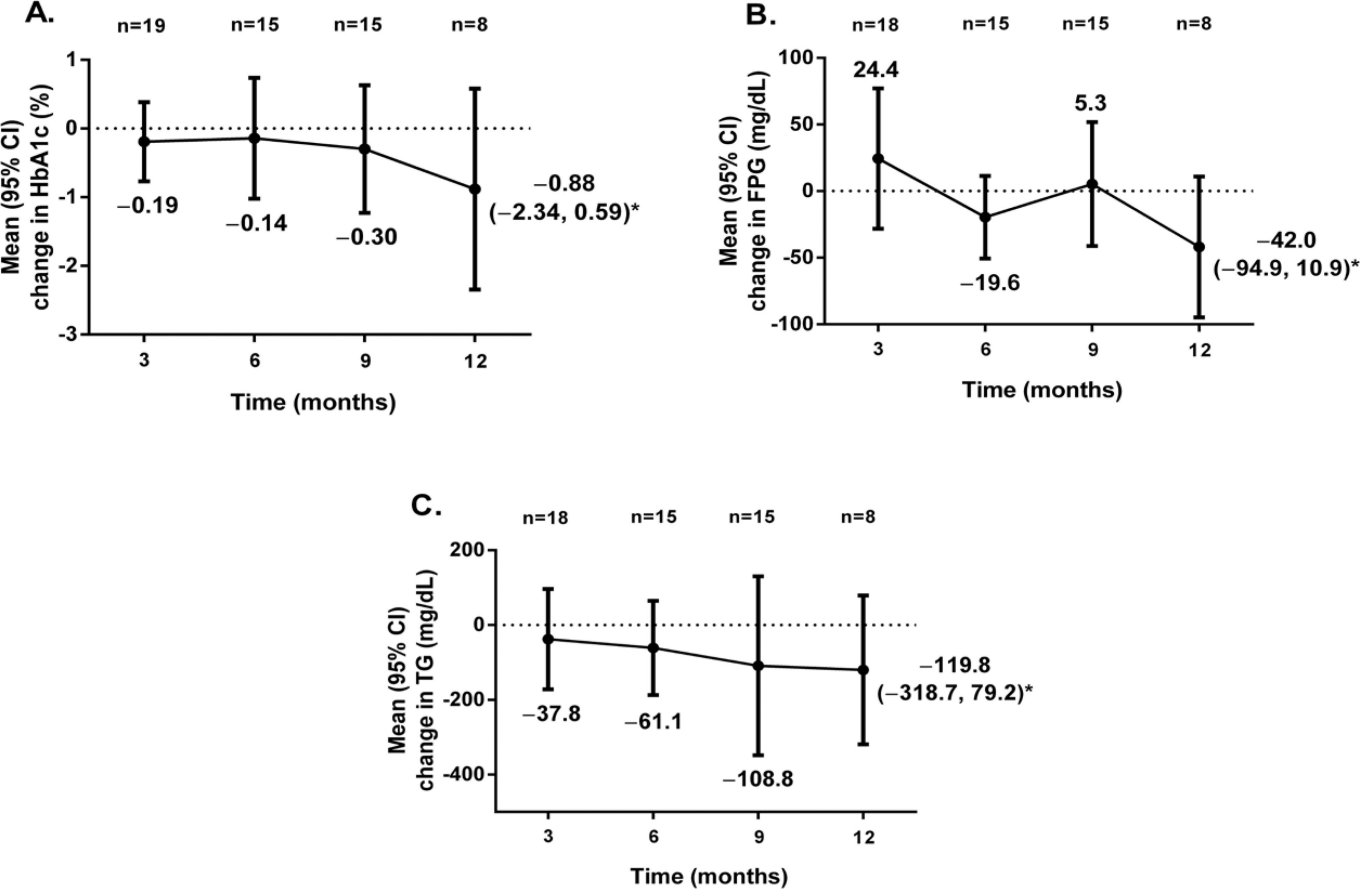
Mean changes from baseline in (**A**) glycated hemoglobin (HbA1c), (**B**) fasting plasma glucose (FPG), and (**C**) triglycerides (TG) in patients with available measurements at each 3-month interval. *Represents the 95% confidence interval (CI) at 12 months.

HbA1c decreased steadily from baseline through 12 months of metreleptin treatment but did not reach statistical significance (Figure 4A). The mean (SE) change in HbA1c at 12 months was −0.88 (0.62)% (95% CI, −2.34, 0.59). FPG was initially increased at 3 months and followed by decreases or no change from baseline at 6 and 9 months (Figure 4B). At 12 months, FPG was reduced from baseline with a mean (SE) change of −42.0 (22.4) mg/dL (95% CI, −94.9, 10.9). Triglycerides followed a similar trend of reduction from baseline over time to that observed with HbA1c (Figure 4C), with a mean (SE) change at 12 months of −119.8 (84.1) mg/dL (95% CI, −318.7, 79.2) approaching statistical significance.

A small subset of patients with greater baseline abnormalities in HbA1c, FPG, and triglycerides experienced numerically greater mean reductions in metabolic parameters after 1 year of treatment compared with all patients with PL. For patients with HbA1c ≥8.0% at baseline and available HbA1c measurements at 12 months (n=5), the mean (SE) change from baseline was −1.44 (0.93)% (95% CI, −4.01, 1.13). Five patients (Patients 1, 4, 8, 10, and 23) appeared to respond well to metreleptin treatment on the basis of their individual HbA1c changes from baseline to last observation (Supplemental Table 2). Doses of oral glucose-lowering medications during the same period were mostly stable (Supplemental Table 1). Metformin dose increased for Patient 23 only, and metformin dosing frequency decreased for Patient 10. Patients 1, 4, 8, and 23 had decreases in their total daily insulin doses. Patient 15 exhibited a marked worsening of HbA1c from 9.0% to 12.9% but was also poorly adherent to baseline medications and metreleptin treatment. For patients with FPG ≥126 mg/dL at baseline and available FPG measurements at 12 months (n=7), the mean (SE) change in FPG was −49.7 (24.2) mg/dL (95% CI, −109.0, 9.6).

The mean (SE) change from baseline for patients with baseline triglycerides ≥200 mg/dL and an available measurement at 12 months (n=5) was −184.4 (127.5) mg/dL (95% CI, −538.3, 169.5). Five patients (Patients 1, 2, 8, 14, and 20) appeared to respond well to metreleptin treatment on the basis of their individual changes from baseline in triglycerides. Specifically, Patient 2 had a reduction from 919 to 275 mg/dL. In the same period, doses of concomitant lipid-lowering medications were stable among Patients 1, 2, 8, 14, and 20. Pravastatin and fish oil were added to prior fenofibrate therapy for Patient 23. Five patients (Patients 12, 15, 17, 18, and 22) exhibited clinically significant worsening of triglycerides from baseline through the end of study visit; of those, Patient 15 was observed to have been consistently nonadherent to therapy. Patient 17 had a normal triglyceride concentration at baseline that was not reflective of historical data prior to the study.

### Safety

**Figure 5:**
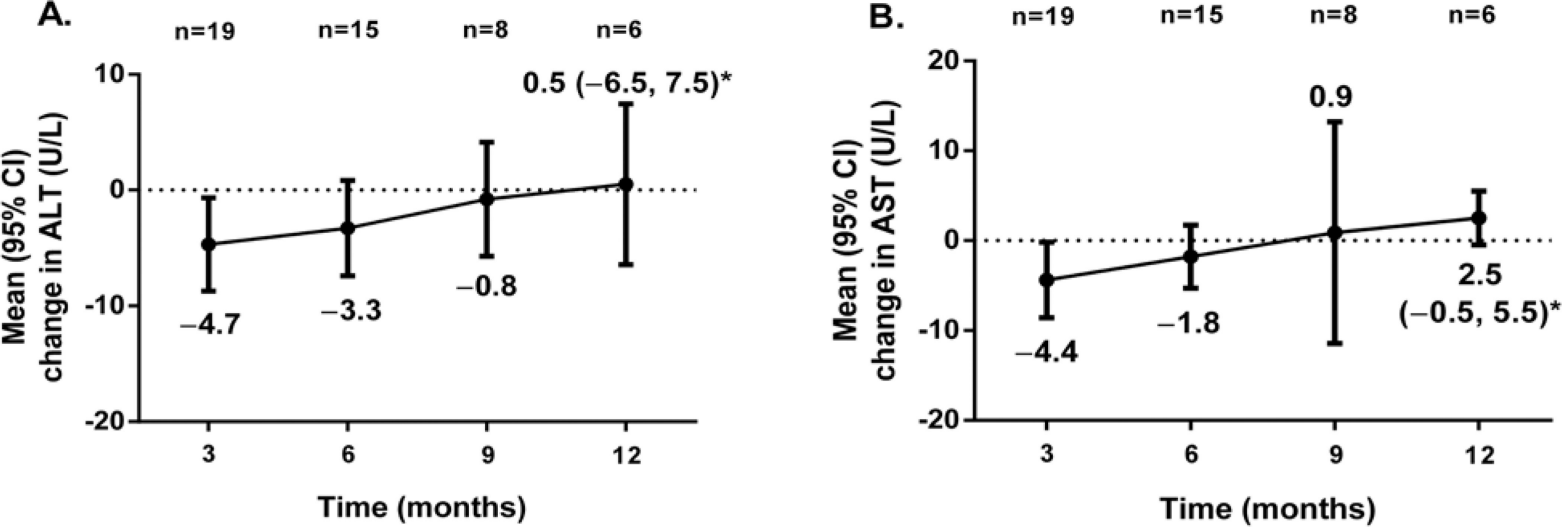
Mean changes from baseline in (**A**) ALT and (**B**) AST in patients with available measurements at each 3-month interval. ALT: Alanine Aminotransferase; AST: Aspartate Aminotransferase. *Represents the 95% confidence interval (CI) at 12 months.

Initial decreases in liver enzymes observed at 3 and 6 months returned back to baseline at 9 months with a small numerical increase at 12 months (Figure 5). Mean (SE) changes in ALT and AST at 12 months were 0.5 (2.7) U/L (95% CI, −6.5, 7.5) and 2.5 (1.2) U/L (95% CI, −0.5, 5.5), respectively. At no time throughout 12 months of treatment were liver enzymes measured at ≥3 times the upper limit of normal.

Most patients (95.7%) experienced a TEAE through 1 year, and most TEAEs were mild or moderate in intensity, with 21.7% experiencing a severe TEAE (Table 2). By preferred term, the most frequently observed TEAEs (≥10% incidence) included nausea (39.1%), hypoglycemia (26.1%), and urinary tract infection (26.1%). Twelve hypoglycemia events were observed in 6 patients with PL. Of these events, 10 were mild in intensity and 1 each was moderate or severe. There were 6 events of urinary tract infection (UTI) occurring in 2 patients with a history of recurrent UTI. All injection site events were mild with the exception of 1 event of injection site pruritus, which was judged to be moderate in intensity.

Eight serious TEAEs occurred in 6 patients (26.1%) and included single events of vertigo, chest pain, cellulitis, *Escherichia coli* UTI, other UTI, gastroenteritis, hypoglycemia, and loss of consciousness. Patients experiencing these AEs usually had a history of similar events prior to treatment. A 67-year-old patient with lipodystrophy who experienced loss of consciousness and resulting subdural hematoma died from a fall. This patient had a history of hypertension, coronary artery disease, peripheral vascular disease, rheumatoid arthritis and systemic lupus erythematosus overlap syndrome, and seizures. There were no reports of treatment-emergent hepatitis, proteinuria, or T-cell lymphoma.

## Discussion

The use of metreleptin is not FDA approved for patients with PL. Based on our experience in this open-label, expanded access program, mean HbA1c, FPG, and triglyceride levels were numerically improved throughout 1 year in the overall cohort, but substantial improvements in a small group of patients may have accounted for the observed result.

The degree of improvement in HbA1c and triglycerides appeared to be greatest among patients with higher abnormal values of these parameters (HbA1c ≥8.0% or triglycerides ≥500 mg/dL) at baseline. This observation is comparable in some aspects with findings from the NIH study in which patients with PL and baseline HbA1c >8.0% or triglycerides >500 mg/dL experienced numerically greater improvements in HbA1c or triglycerides compared with patients with baseline HbA1c >7.0% or triglycerides >300 mg/dL, or compared with all patients with PL [15]. However, it should be clearly stated that our patient population had higher mean baseline leptin (14.8 vs. 6.2 ng/mL) and less severe metabolic abnormalities (lower mean HbA1c [7.9 vs. 8.1%], glucose [141 vs. 182 mg/dL], and triglycerides [402 vs. 483 mg/dL]) compared with patients with PL in the NIH study. By not restricting the inclusion of patients on the basis of leptin levels (NIH study included patients with leptin levels <8 [males] and <12 ng/mL [females]) [15], we had hoped to achieve a greater understanding of the range of patients with PL who would possibly benefit from metreleptin treatment.

In looking at individual patients who had favorable improvements in HbA1c and triglycerides with metreleptin therapy, we believe that earlier onset of disease and evidence for a genetic basis (clear history of Dunnigan pattern of fat loss or confirmed genetic basis) may also be potentially related to better responses in addition to the presence of greater severity of metabolic abnormalities. Further data analyses of all patients with PL in collaboration with other groups reporting data on these syndromes may help to confirm these inferences. Baseline leptin has been shown to be a predictor of triglyceride and HbA1c response to metreleptin [15]; however, in our study, Patients 2, 4, 10, 14, and 20 with clinically significant decreases in HbA1c or triglycerides also had higher baseline leptin values (23.0, 19.1, 17.7, 19.0, and 13.0 ng/mL, respectively). It is important to note that patients who tend to respond well to metreleptin do so within the first few months and typically have sustained efficacy so long as they continue adherence to metreleptin and concomitant medications. We and others have noted temporary worsening of metabolic parameters when patients acutely discontinue or skip doses [13,16,19]. For example, Patient 16 who had elevated triglycerides above baseline at 12 months was actually a responder at 6 months but temporarily skipped doses around the 12-month visit. A repeat value 2 weeks after continuous treatment following this patient’s 12-month visit was 355 mg/dL.

Another important point from our experience is the labile nature of triglyceride levels. We have observed wide fluctuations of triglyceride levels in this patient population. Future trials, especially those seeking regulatory approval of new medications in this patient population, should take into account the dynamic nature of outcome measures such as triglyceride levels and should give consideration to allowing integration of multiple measurements over a prespecified interval. The measured baseline triglyceride value for Patient 17 was not indicative of the patient’s historical values, and their end-of-study measurement suggested metabolic worsening while on metreleptin; whereas, in reality, the patient likely had substantial improvement in their average triglyceride values despite tolerating only low doses of metreleptin. In addition, the patient had clinical improvement in hepatomegaly, weight, and facial fat deposition (Supplemental Figure 2). The baseline state of our patients and those of others reported in the literature underscore that there is still an unmet medical need to treat dyslipidemia in this population. Recently, the use of nutraceuticals and functional foods have emerged as a potentially applicable concept to treat refractory dyslipidemic syndromes [20].

Hepatomegaly, fatty liver, and other liver abnormalities have been reported in other case series of patients with PL [5,8]. In our expanded access experience, 18 patients with PL had a prior history of hepatic steatosis, so it is encouraging that liver enzymes remained mostly stable throughout 1 year of metreleptin treatment and did not worsen. Our findings are similar to those observed from the University of Texas study where patients with PL who were moderately hypoleptinemic saw very little to no change from baseline in ALT and AST after 6 months of metreleptin treatment [13]. An analysis of long-term efficacy and safety from the NIH study also demonstrated substantial reductions in ALT and AST throughout 3 years of metreleptin treatment, but this population included more patients with GL than patients with PL, and mean baseline values were numerically higher compared with our cohort (ALT, 100 vs. 32.3 U/L; AST, 71 vs. 27.8 U/L) [16]. Our study did not quantify hepatic fat, fibrosis by imaging means, or histology, and it is well known that the serum transaminases may not correlate with hepatic steatosis or histopathological findings of steatohepatitis [21].

No new safety concerns were observed throughout 1 year of treatment with metreleptin. The most frequently observed AEs of nausea, hypoglycemia, urinary tract infection, and injection site reactions have been observed in prior experiences with metreleptin therapy [13,15]. The serious TEAEs that were observed did not appear to be related to metreleptin treatment and were usually events that had occurred that were consistent with patients’ past medical histories. It is important to note that the patients in this study had a high disease burden with respect to their underlying conditions, and the open-label design did not allow for comparison of these AEs with a standard treatment or placebo group.

FDA-approved labeling for metreleptin includes a boxed warning on the risks of the development of antibodies with neutralizing activity resulting in increased risk of infection or worsening of metabolic control, and the risk of T-cell lymphoma [11]. Apart from injection site reactions that were mostly mild in intensity, no TEAEs related to immunogenicity were observed. The development of antibodies in response to treatment with therapeutic proteins is commonplace [22], and metreleptin is no exception. In a recent analysis of the immunogenicity of metreleptin across the clinical development program (lipodystrophy and obesity studies), 92% of all patients in the present study developed anti-metreleptin antibodies, but no patients exhibited *in vitro* neutralizing activity of any kind [17]. There were no reports of T-cell lymphoma. Peripheral T-cell lymphoma has been previously reported in patients with acquired GL who have both received and not received metreleptin treatment [23]. The exact mechanism underlying the development of T-cell lymphoma in patients with lipodystrophy has not been fully delineated, but the role of autoimmunity associated with acquired GL has been proposed [23]. We believe that the observed AE profile and supplemental clinical information provided in this paper provides valuable information about the clinical challenges that patients with PL typically face due to their disease state.

Due to the rarity of the disease, only a small number of patients with PL could be evaluated. The modest improvements in metabolic results that were observed should be interpreted with caution given the small size of this study and the lack of a placebo control. Though hypothesis testing was never the goal of this study, post-hoc power analyses reveal almost 80% power only with an alpha error of 0.1 if all patients would have completed the study. With paired data from 8 to 12 patients, the power is <50%.

Additionally, there was no standard algorithm for the management of concomitant medications and clinicians were free to adjust doses and add medications. In some instances, insurance companies dictated medication changes, which is a major obstacle in clinical trials with restricted budgets. It is possible that some treatment benefit observed with metreleptin in select patients may have been due to adjustments made to concomitant medications, although the most common adjustments were decreases in insulin doses; less common was the addition of new medications or increases in current doses. Some of the increases in FPG observed at 3 months post-baseline may have been due to decreases in insulin to avoid hypoglycemia. This study was not designed to measure the effect of metreleptin on hepatic steatosis, liver histopathology, quality of life, fatigue, pain scores, appetite, or sleep apnea. Many patients from this cohort have noted subjective improvement in almost all of these parameters and outcomes. We are very interested in seeing these endpoints evaluated in future trials.

Measures of adherence were not recorded in the present study, and it should be stated that the success of metreleptin depends strongly on patient adherence to the overall treatment plan including dietary modifications, the use of concomitant medications, and metreleptin use itself. Poor adherence has been related to worsened metabolic control in some patients [13,16,19]. Regular patient counseling and education at clinic visits on disease knowledge, proper use of medications, and potential side effects to therapy may be useful strategies for clinicians to help improve adherence in a patient population that has a high need for treatment and medical resources.

In conclusion, Study FHA101 focused on a unique population without any entry criterion for baseline leptin levels, as was the case with previous study groups. Inclusion of patients with mildly low or entirely normal leptin levels allowed us to examine if metreleptin can benefit patients with PL who did not have clear evidence of leptin deficiency but who were having clinical challenges. Looking at individual patients who responded well, we infer that earlier onset of disease, presence of Dunnigan phenotype or other clear genetic abnormalities, and greater severity of baseline diabetes or hypertriglyceridemia are possible indicators of which patients with PL may benefit from metreleptin. An expanded data cut of patients from this study is expected to be completed and may confirm or alter these preliminary findings. Collectively, the modest data from this study and the expanded data cut may be useful to guide future studies and help further refine criteria for which patients with PL may receive the greatest benefits of metreleptin.

## Supplementary Material

Supplementary file

## Figures and Tables

**Table 1:** Baseline demographics.

Baseline characteristics	Patients with partial lipodystrophy (N=23)
**Sex**, n (%)	
Female	22 (95.7)
Male	1 (4.3)
**Race**, n (%)	
White	17 (73.9)
Black	2 (8.7)
Other	4 (17.4)
**Partial lipodystrophy subtype**, n (%)	
Familial partial lipodystrophy	21 (91.3)
Acquired partial lipodystrophy	2 (8.7)
**Age**, years, median (range)	47 (23–67)
**Body weight**, kg, mean (SD)	84.1 (19.2)
**Height**, cm, mean (SD)	163.9 (7.7)
**Body mass index**, kg/m^2^, mean (SD)	31.2 (6.0)
**HbA1c**, %, mean (SD)	7.9 (1.5)
**FPG**, mg/dL, mean (SD)	140.5 (58.6)
**Fasting triglycerides**, mg/dL, mean (SD)	401.9 (537.1)
**ALT**, IU/L, mean (SD)	32.3 (13.1)
**AST**, IU/L, mean (SD)	27.8 (11.7)
**Fasting leptin**, ng/mL, mean (SD)	14.8 (10.3)
**Glucose-lowering medications at baseline**, n (%)	17 (73.9)
**Lipid-lowering medications at baseline**, n (%)	17 (73.9)

ALT: Alanine Aminotransferase; AST: Aspartate Aminotransferase; FPG: Fasting Plasma Glucose; HbA1c: Glycated Hemoglobin; SD: Standard Deviation.

**Table 2:** Incidence (≥10%) of frequently reported TEAEs.

TEAE, n (%)	Patients with partial lipodystrophy (N=23)
**All TEAEs**	22 (95.7)
Mild	19 (82.6)
Moderate	12 (52.2)
Severe	5 (21.7)
**TEAEs by preferred term**	
Nausea	9 (39.1)
Hypoglycemia	6 (26.1)
Urinary tract infection	6 (26.1)
Injection site hematoma	4 (17.4)
Upper respiratory tract infection	4 (17.4)
Vomiting	4 (17.4)
Injection site urticaria	4 (17.4)
Sinusitis	4 (17.4)
Abdominal pain	3 (13.0)
Lymphadenopathy	3 (13.0)
Muscle spasms	3 (13.0)
Myalgia	3 (13.0)

TEAEs: Treatment-emergent Adverse Events.
