# Supplementary material for: Efficacy and Safety of Metreleptin in Patients with Partial Lipodystrophy: Lessons from an Expanded Access Program
Source: J Diabetes Metab. Author manuscript; Available in PMC 2016 Sep 16. (PMC5026130; doi:10.4172/2155-6156.1000659)
Supplement: Supplementary file [file NIHMS780145-supplement-Supplementary_file.pdf]

Supplemental Table 1: Individual patient details and concomitant medications at baseline through 12 months (or last study visit).

No.	Patient information (age/sex, diagnosis, and pertinent past medical history)	Study finished	Concomitant glucose-lowering medications	Concomitant lipid-lowering medications
1	28/F<br>FPL, Dunnigan, *LMNA* mutation confirmed, daughter of patient 8. Diabetes diagnosed in teenage years	Yes	BL: Regular U500 insulin via pump (0.016 mL/hr),* pioglitazone (45 mg QD)<br>6 mo: Regular U500 insulin via pump (0.008 mL/hr), pioglitazone (45 mg QD)<br>12 mo: Regular U500 insulin (0.10 mL BID or TID)	None
2	58/F<br>FPL, Werner phenotype suspected, commercial lipodystrophy panel negative. Diabetes diagnosed at age 24	Yes	BL: Insulin detemir (50 U qAM, 56 U qPM),* insulin aspart (18–24 U TID), metformin (1000 mg BID)<br>6 mo: Insulin detemir (60 U BID), insulin aspart (18–24 U TID), metformin (1000 mg BID)<br>12 mo: Insulin detemir (60 U BID), insulin aspart (18–24 U TID), metformin (1000 mg	BL: Fenofibrate (145 mg QD), atorvastatin (40 mg QD), fish oil (2 g BID)<br>6 mo: Atorvastatin (80 mg q.o.d.), fenofibrate (145 mg QD), fish oil (2 g BID)<br>12 mo: Fenofibrate (145 mg QD), fish oil (2 g BID)

No.	Patient information<br>(age/sex, diagnosis, and pertinent past medical history)	Study finished	Concomitant glucose-lowering medications	Concomitant lipid-lowering medications
			BID)	
3	30/F<br>PL, genetic basis suspected (novel), hypercalciuria, hypomagnesemia, and infertility	Yes	BL: Insulin aspart via pump (90–110 U/day), acarbose (100 mg TID), metformin (1000 mg BID), pramlintide (120 mcg TID)<br>6 mo: Insulin aspart via pump (90–110 U/day), acarbose (100 mg TID), metformin (1000 mg BID), pramlintide (120 mcg TID)<br>12 mo: Insulin aspart via pump (90–110 U/day), acarbose (100 mg TID), metformin (1000 mg BID), pramlintide (120 mcg TID)	BL: Atorvastatin (40 mg QD), fish oil (1 g BID)<br>6 mo: Atorvastatin (40 mg QD), fish oil (1 g BID)<br>12 mo: Atorvastatin (40 mg QD), fish oil (1 g BID)
4	50/F<br>PL, novel syndrome with progressive neurologic manifestations, novel genetic etiology suspected, diabetes onset in teenage years	Yes	BL: Insulin glargine (40 U qHS), insulin aspart (18–42 U TID), metformin (1000 mg BID)<br>6 mo: Insulin glargine (8 U qAM, 26 U qPM), insulin aspart (18–42 U TID),	BL: Fenofibrate (145 mg QD)<br>6 mo: Fenofibrate (145 mg QD)<br>12 mo: Fenofibrate (145 mg QD)

No.	Patient information (age/sex, diagnosis, and pertinent past medical history)	Study finished	Concomitant glucose-lowering medications	Concomitant lipid-lowering medications
			metformin (1000 mg BID)<br>12 mo: Insulin glargine (30 U qPM), insulin aspart (18–42 U TID), metformin (1000 mg BID)	
5	43/F<br>FPL, Type 1 (Köbberling), history of MI during childbirth. Diabetes onset occurred in her 30s	Yes	BL: Insulin detemir (45 U QD), insulin aspart (14–15 U TID), metformin (1000 mg BID)<br>6 mo: Insulin detemir (36 U QD), insulin aspart (14–15 U TID), metformin (1000 mg BID)<br>12 mo: Insulin detemir (30 U qPM), insulin aspart (14–15 U TID), metformin (1000 mg BID)	BL: Simvastatin (20 mg QD)<br>6 mo: Simvastatin (20 mg QD)<br>12 mo: Simvastatin (20 mg QD)
6	31/F<br>PL, severe hyperandrogenism,	No[†]	BL: Regular U500 insulin (0.40 mL BID, 0.30 mL qPM)*	BL: Fenofibrate (145 mg QD)<br>1 mo: Fenofibrate (145 mg QD)

No.	Patient information<br>(age/sex, diagnosis, and pertinent past medical history)	Study finished	Concomitant glucose-lowering medications	Concomitant lipid-lowering medications
	diabetes diagnosis at age 30		1 mo: Regular U500 insulin (0.40 mL BID, 0.30 mL qPM)	
7	67/F<br>APL, Barraquer-Simons variety, significant autoimmune disease history with mixed connective tissue disease and SLE, diagnosis of APL in 30s	No[‡]	BL: Insulin glargine (20 U QD), regular insulin (20 U BID)<br>1 mo: insulin glargine (20 U QD), regular insulin (20 U BID)	BL: Fluvastatin (20 mg QD), fish oil (1 g TID)<br>1 mo: Fluvastatin (20 mg QD), fish oil (1 g TID)
8	57/F<br>FPL, Dunnigan, confirmed *LMNA* mutation, mother of patient 1, affected members suffer from significant CNS pain syndrome and anxiety. Diabetes diagnosed in her 30s	Yes	BL: Pioglitazone (30 mg QD), insulin glargine (30 U qHS)<br>6 mo: Pioglitazone (30 mg QD)<br>12 mo: Pioglitazone (30 mg QD)	BL: Simvastatin (30 mg QD), fish oil (1 g BID)<br>1 mo: Simvastatin (30 mg QD), fish oil (1 g BID)<br>6 mo: Simvastatin (30 mg QD), fish oil (1 g BID)
9	51/F<br>PL, severe insulin resistance,	No[†]	BL: Metformin (1000 mg BID), glimepiride (4 mg QD), pioglitazone (45 mg QD)	None

No.	Patient information<br>(age/sex, diagnosis, and pertinent past medical history)	Study finished	Concomitant glucose-lowering medications	Concomitant lipid-lowering medications
	phenotypically similar to patient 6.<br>Diabetes diagnosed in her 30s		1 mo: Metformin (1000 mg BID), glimepiride (4 mg QD), pioglitazone (45 mg QD)	
10	62/F<br>PL, history of CAD and breast cancer.<br>Diabetes diagnosed in her 30s	Yes	BL: NPH/regular 70/30 insulin (35 U qAM, 65 U qPM), metformin (500 mg BID)<br>6 mo: NPH/regular 70/30 insulin (40 U qAM, 80 U qPM), metformin (500 mg QD)<br>12 mo: NPH/regular 70/30 insulin (30 U qAM, 75 U qPM), metformin (500 mg QD)	BL: Rosuvastatin (40 mg QD)<br>6 mo: Rosuvastatin (40 mg QD)<br>12 mo: Rosuvastatin (40 mg QD)
11	42/F<br>FPL, Dunnigan, *LMNA* mutation confirmed, also *BRCA1* carrier.<br>Diabetes diagnosed in her 20s	Yes	BL: Pioglitazone (30 mg QD)<br>6 mo: Pioglitazone (30 mg QD)<br>12 mo: None	BL: Atorvastatin (10 mg QD)<br>6 mo: Pravastatin (10 mg QD)<br>12 mo: Pravastatin (10 mg QD)
12	23/F<br>PL, subtype unknown, onset around	Yes	BL: Metformin (1000 mg BID, pioglitazone 45 mg QD)	BL: Simvastatin (80 mg QD)<br>6 mo: Simvastatin (80 mg QD)

No.	Patient information (age/sex, diagnosis, and pertinent past medical history)	Study finished	Concomitant glucose-lowering medications	Concomitant lipid-lowering medications
	teenage years with dysmorphic features at birth, severe HTN		6 mo: Metformin (1000 mg BID)<br>12 mo: Metformin (1000 mg BID)	12 mo: Pravastatin (80 mg QD)
13	45/F<br>FPL, Dunnigan, *LMNA* mutation confirmed, anxiety. Diabetes diagnosed at age 19	Yes	BL: Exenatide (10 mcg BID), metformin (500 mg TID), glimepiride (2 mg BID)<br>6 mo: Exenatide (10 mcg BID), metformin (500 mg TID)<br>12 mo: Exenatide (10 mcg BID), metformin (500 mg TID)	BL: Fish oil (1 g QD), colesevelam (625 mg QD)<br>6 mo: Fish oil (1 g QD), colesevelam (625 mg QD)<br>12 mo: Fish oil (1 g QD), colesevelam (625 mg QD)
14	62/F<br>PL, not classic Dunnigan, has autoimmune disease (Still's disease). Diabetes diagnosed in her 30s	Yes	BL: Metformin (500 mg BID), glipizide (10 mg BID), sitagliptin (100 mg QD)<br>6 mo: Metformin (500 mg BID), glipizide (10 mg BID), sitagliptin (100 mg QD)<br>12 mo: Metformin (2000 mg QD), glipizide (10 mg BID), sitagliptin (100 mg QD)	BL: Simvastatin (80 mg QD)<br>6 mo: Simvastatin (80 mg QD)<br>12 mo: Simvastatin (80 mg QD)
15	58/M	Yes	BL: Insulin glargine (140 U BID), insulin	BL: Fish oil (2–3 g/day)

No.	Patient information<br>(age/sex, diagnosis, and pertinent past medical history)	Study finished	Concomitant glucose-lowering medications	Concomitant lipid-lowering medications
	PL, very difficult to control diabetes, clear AD inheritance pattern of fat distribution in the family with a mother and sister affected, BMI in the morbid obesity range. Diabetes diagnosed in 30s		aspart (up to 75 U BID–TID), pramlintide (120 mcg BID)<br>6 mo: Insulin glargine (100 U qPM), insulin aspart (up to 75 U BID–TID), metformin (500 mg, BID), pramlintide (120 mcg TID)[§]<br>12 mo: Glargine insulin (100 U qPM), aspart insulin (30 U BID)	6 mo: Fish oil (2–3 g/day)<br>12 mo: Fish oil (2–3 g/day)
16	40/F<br>APL, Barraquer-Simons, nondiabetic, dyslipidemia is the prominent feature, APL diagnosed at age 19	Yes	None	BL: Fish oil (1 g BID)<br>6 mo: Fish oil (1 g BID)<br>12 mo: Fish oil (1 g BID)
17	55/F<br>FPL, Dunnigan, confirmed *LMNA* mutation, history of elevated triglycerides prominent, but diet	Yes	None	BL: Fish oil (1 g TID)[§]<br>6 mo: None<br>12 mo: Fish oil (1 g BID)[§]

No.	Patient information<br>(age/sex, diagnosis, and pertinent past medical history)	Study finished	Concomitant glucose-lowering medications	Concomitant lipid-lowering medications
	sensitive. Significant myalgia was a prominent symptom. Did not tolerate metreleptin above 2 mg daily			
18	58/F<br>FPL, subtype unknown, clear AD inheritance in the family, also diagnosed with Cushing's disease (likely erroneously), s/p TSS and bilateral adrenalectomy with normal histopathology on specimen from both surgeries. Diabetes diagnosed in her 30s	No[†]	BL: Regular U500 insulin (0.70 mL BID, 0.60 mL qPM)<br>6 mo: Regular U500 insulin (0.20–0.25 mL BID)<br>9 mo: Regular U500 insulin (0.30 mL qAM, 0.20 mL qPM)	BL: Fish oil (2 g BID)<br>6 mo: Fish oil (2 g BID)<br>9 mo: Fish oil (2 g BID)
19	43/F<br>PL, in the FPL spectrum, background myopathy. Muscle biopsy showed	No[†]	None	None

No.	Patient information (age/sex, diagnosis, and pertinent past medical history)	Study finished	Concomitant glucose-lowering medications	Concomitant lipid-lowering medications
	metabolic myopathy, biochemical analyses of muscle tissue suggested phosphofructokinase deficiency, molecular analyses were not confirmatory. Muscle pain led to early withdrawal. Previously statin and fibrate intolerant			
20	40/F<br>FPL, no *LMNA* mutation, severe hyperandrogenism (see Figure 1), fraternal twin of patient 21 and daughter of patient 22	Yes[‖]	None	BL: Simvastatin (40 mg QD)<br>6 mo: Simvastatin (40 mg QD)<br>17 mo: Simvastatin (40 mg QD)
21	40/F<br>FPL, no *LMNA* mutation, severe hyperandrogenism (see Figure 1),	Yes[‖]	None	None

No.	Patient information<br>(age/sex, diagnosis, and pertinent past medical history)	Study finished	Concomitant glucose-lowering medications	Concomitant lipid-lowering medications
	fraternal twin of patient 20 and daughter of patient 22. Patient was statin intolerant			
22	62/F<br>FPL, no *LMNA* mutation, severe hyperandrogenism, mother of patients 20 and 21, significant OSA, CAD, splenomegaly, and thrombocytopenia	Yes#	BL: Regular insulin via pump (2.8 U/hr), regular U500 insulin (1 mL TID)<br>17 mo: Aspart protamine/aspart 70/30 insulin (20 U BID), regular U500 insulin (0.4 mL TID)	BL: Fenofibrate (130 mg QD), rosuvastatin (10 mg weekly)<br>17 mo: Fenofibrate (130 mg QD), rosuvastatin (10 mg weekly)
23	47/F<br>FPL, Dunnigan, diabetes diagnosed in her 20s. Peripheral vascular disease s/p right femoral-popliteal bypass surgery, CAD s/p stenting	Yes	BL: U500 insulin (0.3 mL BID), metformin (500 mg BID)<br>6 mo: U500 insulin (0.2 mL BID), metformin (1 g BID)<br>12 mo: U500 insulin (0.2 mL BID), metformin (1 g BID)	BL: Gemfibrozil (600 mg BID), fish oil (1 g QD)<br>6 mo: Fenofibrate (145 mg QD)<br>12 mo: Fenofibrate (145 mg QD), pravastatin (40 mg QD), fish oil (2 g QD)

AD, autosomal dominant; BID, twice daily; BL, baseline; BMI, body mass index; CAD, coronary artery disease; CNS, central nervous system; F, female; FPL, familial partial lipodystrophy; HTN, hypertension; M, male; MI, myocardial infarction; mo, month(s); NPH, neutral protamine Hagedorn; OSA, obstructive sleep apnea; PL, partial lipodystrophy; qAM, every morning; QD, once daily; qHS, every night at bedtime; q.o.d., every other day; qPM, every evening; s/p, status post; SLE, systemic lupus erythematosus; TID, three times daily; TSS, transsphenoidal surgery; U, units.

[*]Dose reduced at baseline visit to prevent hypoglycemia.

[†]Patient withdrew.

[‡]Patient was deceased during study.

[§]Patient was poorly adherent to metreleptin and/or concomitant medications.

[‖]Patient missed 12-month visit.

[#]Patient missed 6- and 12-month visits.

Supplemental Table 2: Leptin, anthropomorphic measures, metabolic parameters, and liver enzymes at baseline through 12 months (or last study visit).

No.	Age/sex and diagnosis	Study finished	Study visit	Fasting leptin (ng/mL)	Weight (kg)	BMI (kg/m^2)	HbA1c (%)	TG (mg/dL)	TC (mg/dL)	LDL-C (mg/dL)	HDL-C (mg/dL)	AST (U/L)	ALT (U/L)
1	28/F FPL, Dunnigan	Yes	BL:<br>6 mo:<br>12 mo:	8.5	81.3<br>80.1<br>82.7	30.6<br>30.1<br>31.1	8.3<br>7.0<br>6.7	114<br>189<br>89	194<br>211<br>160	124<br>127<br>96	48<br>46<br>46	29<br>24<br>18	43<br>26<br>22
2	58/F FPL, Dunnigan	Yes	BL:<br>6 mo:<br>12 mo:	23.0	83.1<br>81.6<br>82.9	36.8<br>36.2<br>36.8	8.5<br>8.3<br>8.9	919<br>150<br>275	306<br>171<br>186	73<br>106<br>99	33<br>35<br>32	25<br>18<br>25	32<br>20<br>29
3	30/F PL, genetic (novel)	Yes	BL:<br>6 mo:<br>12 mo:	18.4	99.2<br>97.1<br>96.6	39.5<br>38.7<br>38.2	7.0<br>6.9<br>7.0	192<br>186<br>267	141<br>135<br>137	70<br>67<br>49	33<br>31<br>34	21<br>23<br>29	14<br>19<br>26
4	50/F PL	Yes	BL:<br>6 mo:<br>12 mo:	19.1	96.7<br>96.2<br>89.0	36.2<br>36.1<br>33.5	9.1<br>8.2<br>7.2	354<br>267<br>318	253<br>260<br>262	145<br>167<br>160	37<br>39<br>39	20<br>24<br>27	23<br>25<br>28

No.	Age/sex and diagnosis	Study finished	Study visit	Fasting leptin (ng/mL)	Weight (kg)	BMI (kg/m^2)	HbA1c (%)	TG (mg/dL)	TC (mg/dL)	LDL-C (mg/dL)	HDL-C (mg/dL)	AST (U/L)	ALT (U/L)
5	43/F<br>PL	Yes	BL:<br>6 mo:<br>12 mo:	9.5	82.1<br>80.7<br>80.9	27.5<br>27.0<br>27.3	7.9<br>7.9<br>7.8	66<br>83<br>56	177<br>137<br>142	116<br>77<br>91	48<br>43<br>40	20<br>18<br>23	20<br>17<br>23
6	31/F<br>FPL, Dunnigan	No*	BL:<br>1 mo:	N/A	125.7<br>127.9	39.5<br>40.1	7.5<br>N/A	398<br>N/A	169<br>N/A	52<br>N/A	37<br>N/A	30<br>N/A	37<br>N/A
7	67/F<br>APL, Barraquer-Simons (with variation)	No†	BL:<br>1 mo:	N/A	47.2<br>47.3	19.1<br>19.2	8.4<br>N/A	286<br>N/A	187<br>N/A	95<br>N/A	35<br>N/A	21<br>N/A	21<br>N/A
8	57/F<br>FPL, Dunnigan	Yes	BL:<br>6 mo:<br>12 mo:	1.4	57.1<br>56.3<br>59.4	21.0<br>20.7<br>21.8	11.1<br>6.2<br>6.4	193<br>87<br>92	228<br>176<br>215	142<br>99<br>130	48<br>59<br>66	21<br>26<br>22	26<br>18<br>19

No.	Age/sex and diagnosis	Study finished	Study visit	Fasting leptin (ng/mL)	Weight (kg)	BMI (kg/m^2)	HbA1c (%)	TG (mg/dL)	TC (mg/dL)	LDL-C (mg/dL)	HDL-C (mg/dL)	AST (U/L)	ALT (U/L)
9	51/F<br>PL	No*	BL:<br>1 mo:	N/A	106.7<br>106.8	33.1<br>33.1	9.6<br>N/A	94<br>N/A	221<br>N/A	132<br>N/A	70<br>N/A	21<br>N/A	36<br>N/A
10	62/F<br>FPL,<br>Dunnigan<br>suspected	Yes	BL:<br>6 mo:<br>12 mo:	17.7	78.3<br>77.0<br>76.8	31.9<br>31.5<br>31.8	11.0<br>10.9<br>9.0	341<br>108<br>470	165<br>116<br>218	61<br>62<br>99	36<br>33<br>34	21<br>31<br>25	21<br>29<br>35
11	42/F<br>FPL,<br>Dunnigan<br>suspected‡	Yes	BL:<br>6 mo:<br>12 mo:	4.4	66.2<br>64.6<br>62.2	24.0<br>23.4<br>22.6	7.3<br>7.2<br>7.9	253<br>203<br>292	198<br>216<br>227	79<br>110<br>98	69<br>69<br>71	18<br>19<br>19	14<br>15<br>19
12	23/F<br>LD<br>subtype<br>unknown	Yes	BL:<br>6 mo:<br>12 mo:	7	81.5<br>83.6<br>83.6	30.2<br>31.4<br>31.4	6.5<br>6.6<br>6.7	341<br>503<br>614	204<br>208<br>200	101<br>142<br>124	36<br>34<br>37	38<br>28<br>38	46<br>40<br>43
13	45/F	Yes	BL:	6.7	78.0	27.6	7.6	299	148	54	34	27	36

No.	Age/sex and diagnosis	Study finished	Study visit	Fasting leptin (ng/mL)	Weight (kg)	BMI (kg/m^2)	HbA1c (%)	TG (mg/dL)	TC (mg/dL)	LDL-C (mg/dL)	HDL-C (mg/dL)	AST (U/L)	ALT (U/L)
	FPL,<br>Dunnigan[‡]		6 mo:<br>12 mo:		72.7<br>73.0	25.7<br>25.6	7.4<br>7.5	282<br>249	173<br>147	83<br>58	34<br>33	22<br>31	30<br>33
14	62/F<br>FPL,<br>not classic<br>Dunnigan	Yes	BL:<br>6 mo:<br>12 mo:	19.0	79.8<br>79.4<br>74.2	30.2<br>30.4<br>28.1	8.1<br>8.7<br>8.5	304<br>204<br>179	304<br>146<br>132	130<br>73<br>58	39<br>32<br>38	62<br>40<br>30	48<br>35<br>23
15	58/M<br>PL[§]	Yes	BL:<br>6 mo:<br>12 mo:	23.8	117.0<br>114.7<br>113.0	40.8<br>39.9<br>38.6	9.0<br>11.6<br>12.9	108<br>238<br>539	179<br>239<br>298	130<br>156<br>176	27<br>35<br>38	11<br>18<br>16	15<br>23<br>21
16	40/F<br>APL,<br>Barraquer-<br>Simons	Yes	BL:<br>6 mo:<br>12 mo:	8.4	83.1<br>86.5<br>83.3	29.6<br>30.5<br>29.4	5.7<br>5.9<br>5.8	1243<br>399<br>1435	225<br>194<br>313	61<br>82<br>41	23<br>33<br>27	41<br>32<br>37	62<br>52<br>47
17	55/F<br>FPL,	Yes	BL:<br>6 mo:	2.7	57.6<br>50.8	22.5<br>20.0	5.6<br>5.5	79<br>114	178<br>217	127<br>148	36<br>47	18<br>26	18<br>21

**No.**	**Age/sex and diagnosis**	**Study finished**	**Study visit**	**Fasting leptin (ng/mL)**	**Weight (kg)**	**BMI (kg/m^2)**	**HbA1c (%)**	**TG (mg/dL)**	**TC (mg/dL)**	**LDL-C (mg/dL)**	**HDL-C (mg/dL)**	**AST (U/L)**	**ALT (U/L)**
	Dunnigan[‡] [§‖]		12 mo:		51.2	20.1	5.7	150	229	156	42	25	23
18	58/F<br>FPL, unknown type	No*	BL:<br>6 mo:<br>9 mo:	17.9	75.9<br>75.0<br>76.3	31.1<br>32.6<br>33.2	8.2<br>7.7<br>8.4	158<br>177<br>339	246<br>265<br>234	166<br>179<br>116	48<br>50<br>50	53<br>28<br>28	53<br>31<br>36
19	43/F<br>PL, in the FPL spectrum	No*	BL:<br>6 mo:	42.9	83.3<br>84.5	35.7<br>36.2	6.3<br>6.3	201<br>179	237<br>227	42<br>46	154<br>145	25<br>29	39<br>36
20	40/F<br>FPL	Yes	BL:<br>6 mo:<br>17 mo:[#]	13.0	89.7<br>89.5<br>90.0	33.0<br>32.1<br>33.0	7.0<br>7.1<br>7.7	255<br>287<br>155	176<br>172<br>181	84<br>72<br>108	41<br>42<br>42	32<br>27<br>31	41<br>39<br>40
21	40/F<br>FPL	Yes	BL:<br>6 mo:	8.8	84.6<br>85.4	30.0<br>30.4	5.8<br>5.8	249<br>281	243<br>254	144<br>140	49<br>58	23<br>15	34<br>17

No.	Age/sex and diagnosis	Study finished	Study visit	Fasting leptin (ng/mL)	Weight (kg)	BMI (kg/m^2)	HbA1c (%)	TG (mg/dL)	TC (mg/dL)	LDL-C (mg/dL)	HDL-C (mg/dL)	AST (U/L)	ALT (U/L)
			17 mo:[#]		87.2	30.7	6.2	226	293	191	57	21	34
22	62/F<br>FPL	Yes	BL:<br>17 mo:**	28.3	112.7<br>108.5	38.5<br>38.2	6.8<br>7.3	257<br>403	257<br>249	177<br>119	29<br>34	29<br>20	26<br>17
23	47/F<br>FPL,<br>Dunnigan	Yes	BL:<br>6 mo:<br>12 mo:	N/A	68.0<br>73.0<br>73.0	28.8<br>30.9<br>30.9	10.4<br>N/A<br>8.0	899<br>N/A<br>666	218<br>N/A<br>203	N/A<br>N/A<br>N/A	28<br>N/A<br>34	75<br>N/A<br>40	76<br>N/A<br>40

ALT, alanine aminotransferase; APL, acquired partial lipodystrophy; AST, aspartate aminotransferase; BL, baseline; BMI, body mass index; F, female; FPL, familial partial lipodystrophy; HbA1c, glycated hemoglobin; HDL-C, high-density lipoprotein cholesterol; LD, lipodystrophy; LDL-C, low-density lipoprotein cholesterol; M, male; mo, month(s); N/A, not available; PL, partial lipodystrophy; TC, total cholesterol; TG, triglycerides

*Patient withdrew.

[†]Patient was deceased during study.

[‡]In addition to clinically significant weight loss, the patient exhibited improvement in facial fat deposition.

[§]Patient was nonadherent to medication.

[ǀ]Patient exhibited clinical improvement in hepatomegaly.

[#]Patient missed 12-month visit.

**Patient missed 6- and 12-month visits.

Supplemental Figure 1: Baseline proportions of patients using medical therapy for (**A**) diabetes and (**B**) hypertriglyceridemia.

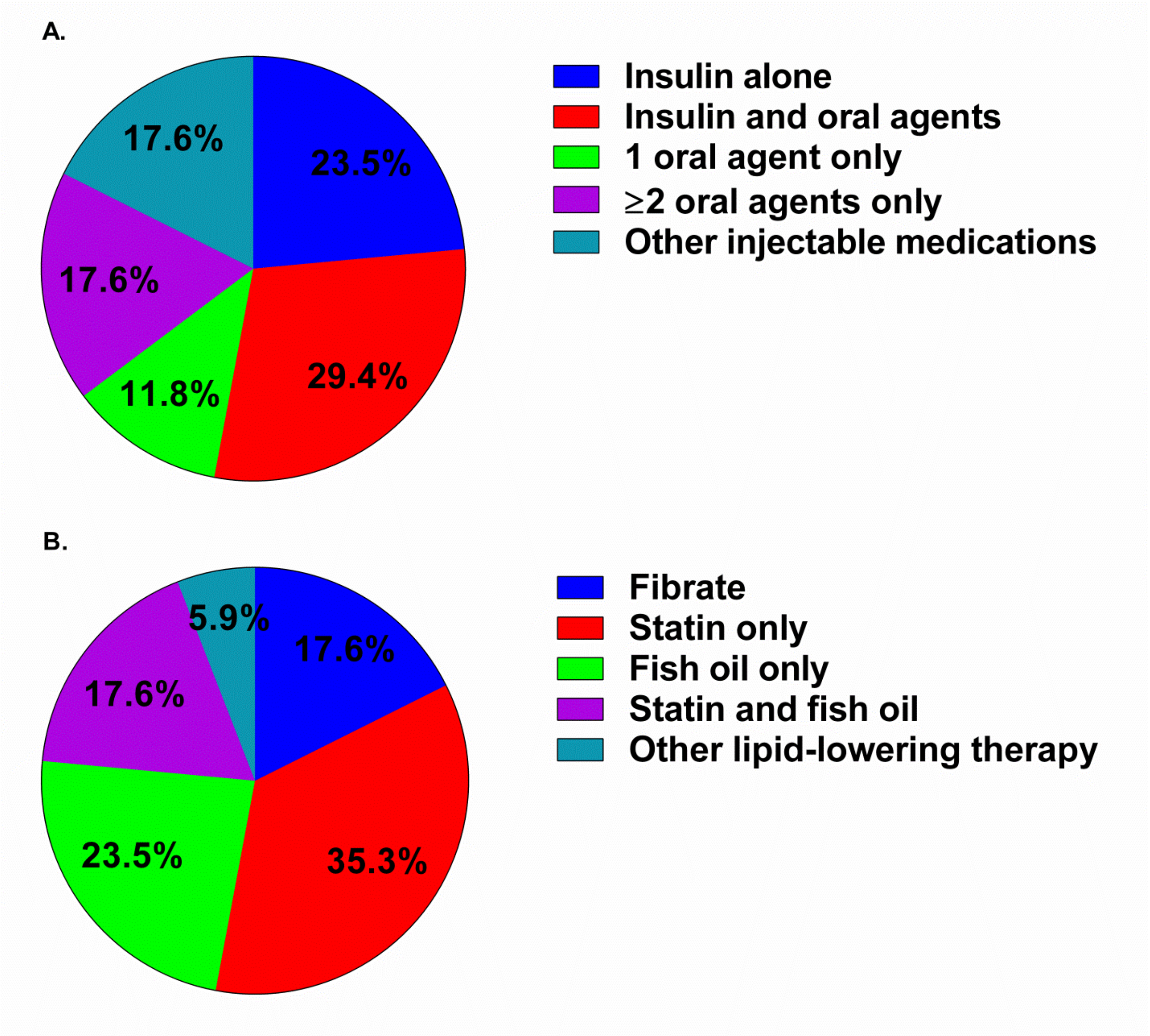

Supplemental Figure 2: Improvement in facial fat deposition in Patient 17 treated with metreleptin.

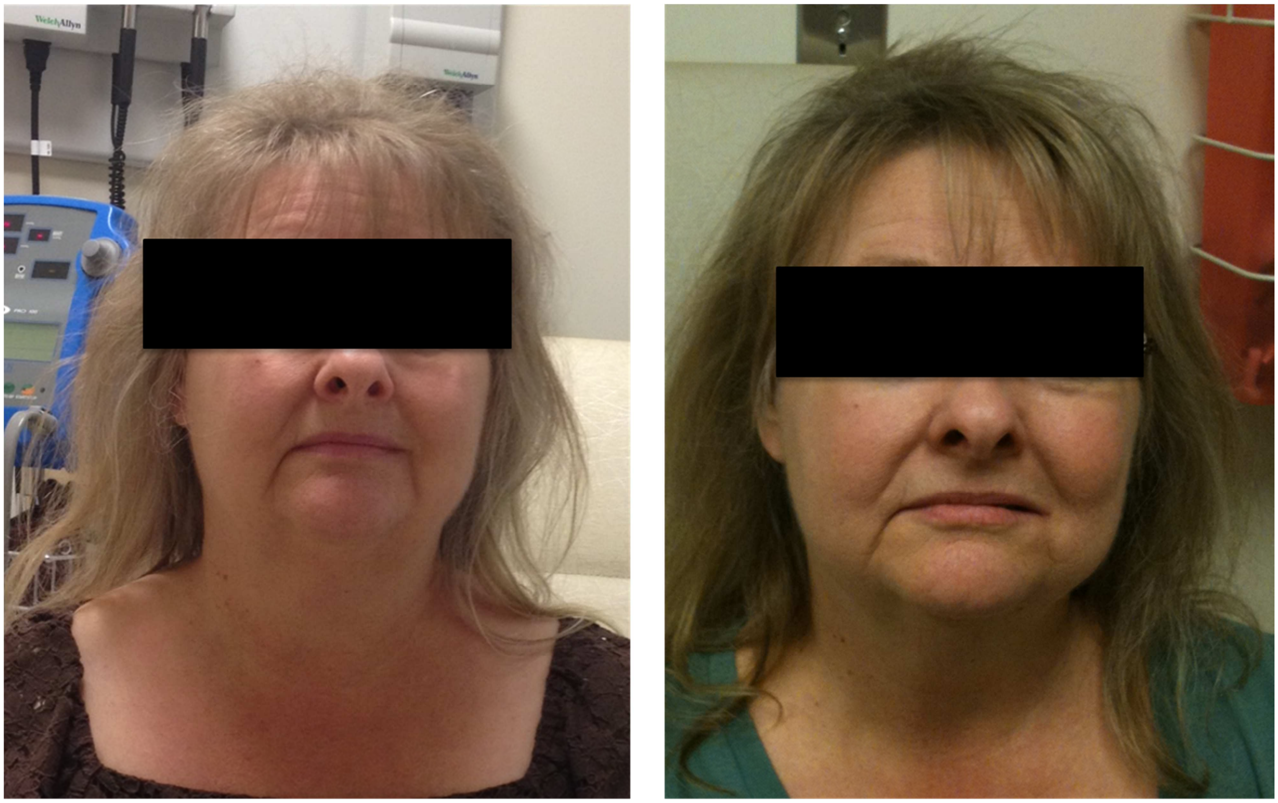

Off Metreleptin   On Metreleptin
